# Characterization of hospital airborne SARS-CoV-2

**DOI:** 10.1186/s12931-021-01637-8

**Published:** 2021-02-26

**Authors:** Rebecca A. Stern, Petros Koutrakis, Marco A. G. Martins, Bernardo Lemos, Scot E. Dowd, Elsie M. Sunderland, Eric Garshick

**Affiliations:** 1 Harvard John A. Paulson School of Engineering and Applied Science, Harvard University, Cambridge, MA USA; 2 Department of Environmental Health, Harvard T.H. Chan School of Public Heath, Boston, MA USA; 3 Department of Environmental Health and Molecular and Integrative Physiological Sciences Program, Harvard T.H. Chan School of Public Health, Boston, MA USA; 4 Molecular Research LP (MR DNA), Shallowater, TX USA; 5 Pulmonary, Allergy, Sleep, and Critical Care Medicine Section, VA Boston Healthcare System, 1400 VFW Pkwy, West Roxbury, Boston, MA 02132 USA; 6 Channing Division of Network Medicine, Brigham and Women’s Hospital, Boston, MA USA; 7 Harvard Medical School, Boston, MA USA

**Keywords:** COVID-19, SARS-CoV-2, Aerosol, Particulate matter, Size fraction

## Abstract

**Background:**

The mechanism for spread of SARS-CoV-2 has been attributed to large particles produced by coughing and sneezing. There is controversy whether smaller airborne particles may transport SARS-CoV-2. Smaller particles, particularly fine particulate matter (≤ 2.5 µm in diameter), can remain airborne for longer periods than larger particles and after inhalation will penetrate deeply into the lungs. Little is known about the size distribution and location of airborne SARS-CoV-2 RNA.

**Methods:**

As a measure of hospital-related exposure, air samples of three particle sizes (> 10.0 µm, 10.0–2.5 µm, and ≤ 2.5 µm) were collected in a Boston, Massachusetts (USA) hospital from April to May 2020 (*N* = 90 size-fractionated samples). Locations included outside negative-pressure COVID-19 wards, a hospital ward not directly involved in COVID-19 patient care, and the emergency department.

**Results:**

SARS-CoV-2 RNA was present in 9% of samples and in all size fractions at concentrations of 5 to 51 copies m^−3^. Locations outside COVID-19 wards had the fewest positive samples. A non-COVID-19 ward had the highest number of positive samples, likely reflecting staff congregation. The probability of a positive sample was positively associated (*r* = 0.95,* p* < 0.01) with the number of COVID-19 patients in the hospital. The number of COVID-19 patients in the hospital was positively associated (*r* = 0.99,* p* < 0.01) with the number of new daily cases in Massachusetts.

**Conclusions:**

More frequent detection of positive samples in non-COVID-19 than COVID-19 hospital areas indicates effectiveness of COVID-ward hospital controls in controlling air concentrations and suggests the potential for disease spread in areas without the strictest precautions. The positive associations regarding the probability of a positive sample, COVID-19 cases in the hospital, and cases in Massachusetts suggests that hospital air sample positivity was related to community burden. SARS-CoV-2 RNA with fine particulate matter supports the possibility of airborne transmission over distances greater than six feet. The findings support guidelines that limit exposure to airborne particles including fine particles capable of longer distance transport and greater lung penetration.

## Background

The rapid spread of coronavirus disease 2019 (COVID-19) raises questions about guidelines regarding droplet and aerosol exposure control measures. Recent studies emphasize the potential for airborne transmission [1–5]. However, there is ongoing debate about the potential for aerosol transmission of the disease and the particle size responsible for it. Larger particles are generated by coughing [6] and sneezing [7], while smaller particles are emitted during speaking and formed by secondary processes such as particle aging or evaporation [8]. Smaller particles remain airborne for longer periods of time and may travel farther than the six-foot (1.83 m) separation distance recommended during the current pandemic.[9] Fine particles ≤ 2.5 µm penetrate deeply into the lungs, particles 10.0–2.5 µm mainly deposit in the larger tracheal-bronchial airways, and particles > 10.0 µm deposit in the upper respiratory tract. The size of the particle impacts the likelihood of infection by inhaled pathogens [10].

Severe acute respiratory syndrome coronavirus 2 (SARS-CoV-2) [11], the virus that causes COVID-19, has been found to be viable in the air [12]. However, there have been limited efforts to identify the size fraction of particulate matter (PM) associated with airborne SARS-CoV-2. In hospital patient areas and a medical staff office in Wuhan, China, three samples were collected into distinct size fractions (> 2.5 µm, 2.5–1.0 µm, 1.0–0.5 µm, 0.50–0.25 µm, and < 0.25 µm) [13]. The authors found SARS-CoV-2 RNA was associated with smaller size fractions near protective apparel removal rooms and with larger sizes in the medical staff office. In Singapore, three size-fractionated samples were collected in a patient’s room with SARS-CoV-2 RNA detected in 1 to 4 µm and > 4 µm size fractions [14]. These findings suggest that SARS-CoV-2 RNA may be found in aerosols in hospital areas near and where COVID-19 patients receive care.

Determining the size of particles carrying viral RNA is critical to understanding their respiratory tract deposition, health impact, residence time in ambient air, and the potential for longer distance transport. Other than one study conducted inside the Nebraska Biocontainment/National Quarantine Unit [15], air monitoring has not been conducted in a U.S. hospital caring for COVID-19 patients. Little is known about the presence of the virus in hospital areas that are not directly involved in known COVID-19 patient care. There is no experimental data regarding the effectiveness of airborne control measures instituted by hospitals in response to the pandemic.

Boston was one of the first U.S. cities to be severely impacted by COVID-19 in 2020. This study was conducted at the Veteran’s Affairs (VA) Boston Healthcare System in West Roxbury, Massachusetts, USA, a medium-sized hospital (134 staffed beds during the study) and the major VA medical center in the Boston area. To deal with the large influx of COVID-19 patients, existing wards were converted entirely to negative pressure ventilation areas requiring full personal protective equipment (PPE) to enter. Hospital locations nearby and outside these isolation units were heavily trafficked by staff. It is important to investigate whether these isolation procedures were effective in eliminating airborne SARS-CoV-2 RNA in neighboring hospital areas. In this study, for the first time, we simultaneously collected airborne particles of three size ranges, > 10.0 µm, 10.0–2.5 µm, and ≤ 2.5 µm, in an acute care hospital environment in locations outside of COVID-19 patient care areas and in non-COVID wards to examine the size of particles and locations associated with SARS-CoV-2 RNA.

## Methods

### Collection design

**Fig. 1:**
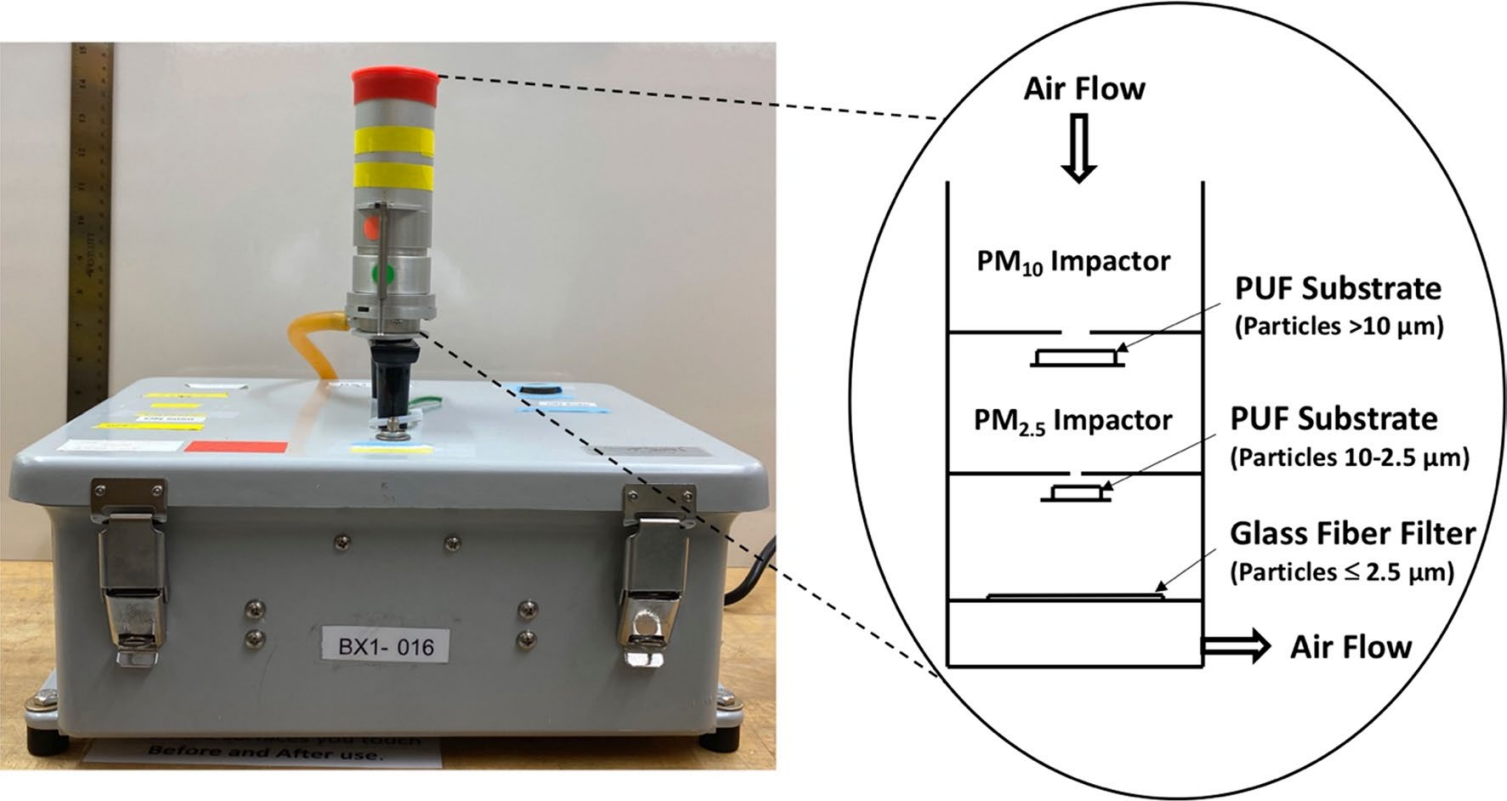
Micro-environmental cascade impactor designed and custom-built by the Environmental Chemistry Laboratory at the Harvard T.H. Chan School of Public Health (HSPH). *PUF* polyurethane foam

We used a micro-environmental cascade impactor designed and custom-built by the Environmental Chemistry Laboratory at the Harvard T.H. Chan School of Public Health (Fig. 1) [16]. This sampler simultaneously collects airborne particles in three size ranges. Large particles (> 10.0 µm) and coarse particles (10.0–2.5 µm) are collected on polyurethane foam (PUF) impaction substrates. Fine particles (≤ 2.5 µm) are collected on a 37-mm glass fiber filter (GFF). The box contains a pump (VP0125, Medo, USA) that provides a constant flow rate of 5 L per minute. Additional file 1: section “Sample collection details” includes additional protocol information.

### Setting and sampling scheme

Five sites were sampled simultaneously (Table 1) six times from April 29 through May 22, 2020. Each sample ran for 48 h at a constant flow rate of 5 L/min for a total volume of 14.4 m^3^ per sampling period. Samplers were located: (1) outside the entrance to a COVID-19 ward (CW1); (2) in a personal protective equipment (PPE) donning room outside the entrance to another COVID-19 ward (CW2); (3) outside the entrance to the medical intensive care unit (ICU); (4) at a staff workstation in the emergency department (ED); and (5) at a nursing staff workstation of a ward not designated for care of COVID-19 patients (NCW) (Table 1). CW1 was closed for cleaning from May 12–18, 2020 but sampling continued throughout this period. Additional details of the sampling locations are provided in Additional file 1 section “Sampling location details”. Anyone entering the ICU, CW1, and donning PPE in CW2 passed in proximity to the sampler because these three locations were at the entry points to the patient care units. Hospital policy during the sampling period included: universal masking for staff and patients when outside their rooms, restricted visitation, and universal admission testing during the time period of the study.

**Table 1:** Sampling locations

Location code	Brief description	Details
ED	Emergency department provider workstation	Provider computer workstation across from two-negative pressure rooms used for suspect COVID-19 patients
ICU	Outside entrance door to COVID-19 ICU	In a corridor outside main entrance to the COVID-19 medical ICU
CW1	Corridor outside COVID-19 ward entrance	Midway through the study (May 12–18, 2020), this location was closed and cleaned; vacant on May 19; and later opened May 20 as a non-COVID-19 ward that includes a smaller unit that cared for suspected COVID-19 patients
CW2	PPE donning room outside COVID-19 ward	PPE donning room that exits into a corridor to a second COVID-19 medical ward
NCW	Nursing workstation in non-COVID-19 ward	Open work area with computer workstation to chart patient notes and exchange information at shift changes

*ED* emergency department, *ICU* intensive care unit, *CW1* COVID-19 Ward 1, *CW2* COVID-19 Ward 2, *NCW* non-COVID-19 Ward

The cascade inlets were located approximately at breathing zone height, 48 to 56 inches above the floor. Field blanks were used and processed simultaneously with the samples. Blanks were taken to the hospital together with the samples but were not exposed to air flow.

### Processing

After each collection, the substrates were removed aseptically from the cascades, placed individually inside 5-mL sterile centrifuge tubes, immersed in RNAlater Stabilization Solution (Ambion, Inc., Austin, TX, USA), and stored in sterile Whirl-Pak (Whirl-Pak, Nasco, USA) bags at 4 °C.

### Sample analysis

Samples and blanks were shipped overnight on ice to Molecular Research DNA (Shallowater, TX, USA), where RNA extraction and reverse transcription quantitative polymerase chain reaction (RT-qPCR) were performed. Viral RNA was extracted using the RNeasy Mini Kit (Qiagen, Hilden, Germany) following the manufacturer's instructions. 5.6 µg Poly-A carrier RNA (Qiagen, Hilden, Germany) was also mixed with each sample for extraction. Carrier RNA enhances the low copy viral nucleic acids binding to the mini column and also reduces the chance of viral RNA degradation. RNA was eluted in 40 µl RNase free water. RNA quantity and quality were determined using NanoDrop2000 (Thermo Scientific, Waltham, MA, USA). Samples were then used to quantify the viral concentrations by qPCR using 2019-nCoV CDC qPCR probe assays (Integrated DNA Technologies, Inc., Coralville, IA, USA) for the nucleocapsid N gene (Additional file 1: Table S1). 12 µl of RNA sample was used for cDNA synthesis using QuantiTect Reverse Transcription kit (Qiagen, Hilden, Germany). 2 µl of the synthesized cDNA was used to perform the qPCR reaction using 2X PrimeTime Gene Expression Master Mix (Integrated DNA Technologies, Inc., Coralville, IA, USA) in the StepOnePlus Real-Time PCR System (Applied Biosystems, Waltham, MA, USA). The qPCR reaction was carried out with an initial holding stage of 95 °C for 3 min for PCR enzyme activation. The cycling stage consisted of 45 cycles of 95 °C for 5 s, followed by 55˚C for 30 s. Genomic RNA from SARS-CoV-2 (2019-nCoV/USA-WA1/2020; ATCC, Manassas, VA, USA) was used as a standard. Positive samples were identified as those with a cycle threshold cutoff of 40.85 that corresponded to one copy number. One sample was selected at random for shotgun sequencing to further evaluate SARS-CoV-2 RNA. The section “Shotgun Sequencing” in Additional file 1 describes complete methods and clade assignments. Genome sequences were submitted to GenBank [17] (accession number MW047086) and analyzed using NextStrain [18].

### Data analysis

The method for calculating air concentration (copy number per m^3^) is provided in Additional file 1: Figure S1. The probability of a positive sample for each sampling period was calculated as the number of positive size-fractionated samples divided by 15 (the number of size-fractionated samples collected per sampling period). Pearson correlation analysis was used to assess the association between the number of hospitalized COVID-19 patients and the probability of detecting a positive sample and new cases in Massachusetts [19] (averaged over the sampling dates). Associations were also assessed between the probability of a positive sample in the ED and number of ED patients, including those with respiratory complaints.

## Results

### Air concentrations of SARS-CoV-2

Table 2 presents the air concentrations (copies m^−3^) of SARS-CoV-2 RNA in five areas of the hospital over six sampling periods per location. Concentrations ranged from 5 to 51 copies m^−3^, with an overall rate of positive samples of 9% of the 90 size-fractionated samples. All field and laboratory blanks were negative for SARS-CoV-2 RNA by PCR. The highest concentrations were observed in the emergency department (ED) on May 13–15 at 51 copies m^−3^ (Table 2). The second highest concentration occurred at the non-COVID-19 ward nurse’s station (NCW) on May 11–13 at 47 copies m^−3^.

**Table 2:** Concentration of SARS-CoV-2 (copies m^−3^) for each 48-h sampling period starting the morning of the start date

Dates	Size	ED	ICU	CW1	CW2	NCW	Probability of positive sample	Average number of COVID-19 patients in hospital
(start–end)								
29 April–1 May	F	0	7	0	0	0	3/15	33
	C	0	0	0	0	0		
	L	0	5	0	0	12		
5 May–7 May	F	0	0	0	0	0	2/15	24
	C	8	0	0	0	5		
	L	0	0	0	0	0		
11 May–13 May	F	0	0	0	0	0	2/15	17
	C	0	0	9	0	0		
	L	0	0	0	0	47		
13 May–15 May	F	51	0	0	0	0	1/15	14
	C	0	0	0	0	0		
	L	0	0	0	0	0		
18 May–20 May	F	0	0	0	0	0	0/15	9
	C	0	0	0	0	0		
	L	0	0	0	0	0		
20 May–22 May	F	0	0	0	0	0	0/15	7
	C	0	0	0	0	0		
	L	0	0	0	0	0		
Number of positive samples		2	2	1	0	3		
Total number of samples (size fractions)		18	18	18	18	18		

*ED* emergency department, *ICU* intensive care unit, *CW1* COVID-19 Ward 1, *CW2* COVID-19 Ward 2, *NCW* non-COVID-19 Ward, *F* fine, ≤ 2.5 µm; *C* coarse, 2.5–10 µm; *L* large, > 10 µm

Designation as a COVID-19 ward was not associated with a greater prevalence of positive samples. The location with the highest prevalence of positive samples was the NCW (17%) (Table 3). The locations with COVID-19 patients—COVID-19 Ward 1 (CW1), COVID-19 Ward 2 (CW2), and intensive care unit (ICU)—had the lowest prevalence of positive samples (6% combined). CW1 had only one positive sample, and it did not occur when the ward was used for patient care, but rather when the ward was closed and being cleaned. CW2 was the only location without any positive samples. The ED staff workstation had a prevalence of positive samples equal to 11%.

**Table 3:** Number of positive samples detected in each location by size fraction

	Fine (≤ 2.5 µm)	Coarse (10.0–2.5 µm)	Large (> 10.0 µm)	Total
ED	1	1	0	2
ICU	1	0	1	2
CW1	0	1	0	1
CW2	0	0	0	0
NCW	0	1	2	3
Total	2	3	3	8

*ED* emergency department, *ICU* intensive care unit, *CW1* COVID-19 Ward 1, *CW2* COVID-19 Ward 2, *NCW* non-COVID-19 Ward

### Particle size association

Viral RNA was detected in all size fractions with about the same frequency (Table 3). The ED had positive samples in the fine (≤ 2.5 µm) and coarse (10.0–2.5 µm) particle size fractions. Outside the ICU, SARS-CoV-2 RNA was detected in the fine and large (> 10.0 µm) size fractions. Positive samples from the NCW were found in the coarse and large size fractions. The greatest concentration (51 copies m^−3^) occurred in the fine size fraction in the ED. The second greatest concentration (47 copies m^−3^) occurred in the large size fraction in the NCW.

### Association with COVID-19 patients

There was a significant positive association between the probability of detecting a positive sample and the average number of COVID-19 patients in the hospital during each sampling period (*r* = 0.95,* p* < 0.01). The number of COVID-19 cases in the hospital was positively associated with the number of new COVID-19 cases in Massachusetts averaged over the corresponding sampling period (*r* = 0.99,* p* < 0.01). The two greatest concentrations occurred on May 11–13 and May 13–15 when COVID-19 patient density in the hospital was not at its highest (Table 2). There was no association between the probability of a positive sample in the ED and the number of patients in the ED or the number of patients evaluated with respiratory complaints in the ED (Table S2). The only positive samples outside the ICU occurred during the sampling period from April 29-May 1, when the hospital COVID-19 burden was at its highest (Table 2)*.*

## Discussion

Although SARS-CoV-2 RNA was present in 9% of all samples, no positive samples were found in the vicinity of CW1 or CW2 while they were used for patient care. The only positive sample in CW1 occurred while it was closed for cleaning. During this time, the negative pressure exhaust system was no longer in use, the ward doors were open, and cleaning crews were passing by the sampler to sanitize the ward. Other studies have documented positive air samples collected in COVID-19 patient rooms [13–15]. Our finding of no viral RNA outside of the COVID-19 wards while they were active (and only one positive sample outside the ICU) suggests that the negative pressure units were effective in limiting airborne exposure outside the units. Previous studies have shown that inside COVID-19 wards, including inside patient rooms, airborne SARS-CoV-2 RNA is detectable [13–15].

Unexpectedly, the nurses’ station on the non-COVID 19 ward (NCW) had the greatest number of positive samples. We observed frequent congregation of staff and consultants at this location. Although it was policy for all hospital personnel to wear masks, it is possible that the positive samples were due to breaches of mask-wearing. The lack of association between the number of patients in the ED and probability of a positive sample in the ED may be due to the fact that the patients in the ED were not predominantly COVID-19 patients, and the positive samples may instead reflect staff activity and patient flow near the ED workstation. The finding of greater positive rates in non-COVID-19 locations, in conjunction with the positive association between probability of a positive sample across all locations and the number of COVID-19 patients in the hospital, suggests that presence of SARS-CoV-2 RNA in the hospital reflects the disease burden more broadly in the community. This conclusion is supported by the strong positive association between the number of COVID-19 patients in the hospital and average daily new cases in Massachusetts.

The fact that we found concentrations in all particle size fractions suggests that virus-containing particles are from sources at different proximities to the sampler or produced by different mechanisms. SARS-CoV-2 RNA on larger particles, such as those in the NCW, may have been due to a cough by someone located near the sampler. Coughing generates larger particles than speaking [20]. Viral RNA that was associated with smaller particles, such as that found in the ED, may reflect a greater distance between the sampler and source, formation of smaller aerosols from larger droplets (e.g., by evaporation), or production by processes emitting smaller particles (e.g., speaking as opposed to coughing). Finding positive samples during the cleaning period in CW1 may be due to resuspension caused by cleaning. We found these particles in the coarse (10.0–2.5 µm) size fraction. Liu et al. (2020) suggested that lofting of coarse particles may be caused by resuspension of particles from floors and hard surfaces [13].

This is the first study to document the presence of SARS-CoV-2 RNA in size-fractionated air samples in non-COVID-19 areas in a U.S. hospital. Previous efforts to study COVID-19 have been focused in COVID-19 patient care areas, with samplers located close to the source (infected patients) and collected onto a single bulk filter to analyze total suspended particulate (TSP). Santarpia et al. (2020), Ong et al. (2020), and Ding et al. (2020) all collected only TSP [15, 21, 22]. Liu et al. (2020) collected samples in Wuhan, China that were mostly TSP, with only three size-segregated samples [13]. Chia et al. (2020) also collected only three size-segregated samples [14]. We detected maximum concentrations on the same order of magnitude as the size segregated samples of Liu et al. (51 copies m^−3^ in our study; 42 copies m^−3^ for Liu et al., 2020) [13]. Liu et al. (2020) found higher concentrations of SARS-CoV-2 RNA in the fine PM fraction than in larger sizes [13]. Chia et al. (2020) had positive samples in the 4–1 µm size fractions [14]. These results support our finding of SARS-CoV-2 RNA associated with fine particles that are capable of long-distance transport.

We detected a greater percentage of positive samples compared to some previous studies. The percent of positive samples was greater in our study (9%) than in the study by Ong et al. (2020) (0%) and Ding et al. (2020) (2%), despite the fact that these studies were conducted in COVID-19 patient care areas [21, 22]. Potential explanations for our higher positive sample rate may be related to a greater viral load in the air or methodological differences. For instance, our study collected a greater volume of air per sample (14.4 m^3^) compared to these studies (1.2 and 1.0 m^3^, respectively) and more samples.

The percentage of positive samples was smaller in our study compared to Chia et al. (2020) (67%), Liu et al. (2020) (77%), and Santarpia et al. (2020) (58–63%).[13, 14, 15]. A possible explanation is the proximity to the source: Chia et al. collected only in airborne infection isolation rooms of COVID-19 patients [14], and Santarpia collected only inside the Nebraska Biocontainment/National Quarantine Unit [15]. Liu et al. sampled under conditions of higher disease prevalence (in Wuhan in February and March, 2020) [13]. Differences in extraction efficiency from the collection substrate, variability in RNA degradation rates, or differences in PCR sensitivity among studies may also explain the differences in rates of positives samples and air concentrations.

The estimation of airborne virus concentrations (copies m^−3^) assumes that there is a continuous emission source. However, it is more likely that the emissions of the virus occurred as isolated events (e.g., a sneeze, cough, or speaking) from infected people rather than as a continuous flux over the entire 48-h sampling period. Since the calculated concentrations are time-weighted averages, someone exposed at the time of emission would likely receive a larger dose over a shorter time period than those implied based on the calculated concentrations.

While the present study detected SARS-CoV-2 RNA in hospital air samples, it did not determine whether the airborne virus was viable (capable of causing infection). Lednicky et al. (2020) recently reported that SARS-CoV-2 in hospital air is infectious [12]. Santarpia et al. (2020) found viable SARS-CoV-2 in particles < 1 µm [23]. Laboratory-generated aerosols containing SARS-CoV-2 were found to remain infectious for three [24] to 16 h [25]. The infectious dose of SARS-CoV-2 is still unknown. It is possible that the infectious dose of SARS-CoV-2 is similar to that of SARS-CoV-1 [26], which was estimated to require 280 viral particles to cause illness in 50% of people [27]. The concentrations measured in the present study are likely underestimated, which may be attributable to losses during extraction from the substrates, RNA degradation, and the sensitivity of PCR, as we detected SARS-CoV-2 RNA by shotgun sequencing in a sample near the lower limit of PCR detection (see section “Shotgun Sequencing” in Additional file 1).

## Conclusion

The COVID-19 pandemic has challenged preconceptions about virus transmission. Our findings support changes in guidance from international bodies including the World Health Organization that help prevent airborne transmission of the virus. The findings promote universal masking for patients and providers and social distancing, even in non-COVID-19 hospital areas, to prevent future spread during this pandemic. Hospital policies such as construction of negative pressure controls in existing wards appear to be effective in reducing airborne concentrations of the virus. The fact that positive samples were concentrated in regions of the hospital with greater congregation of personnel indicates that airborne viral RNA exposure most likely occurs where the concentration of humans is greatest, regardless of whether or not those areas are dedicated for COVID-19 patient care. It suggests that greatest risk of airborne transmission occurs in areas that are treated socially or anecdotally as less risky with respect to COVID-19 exposure. Presence of the virus with fine particles highlights the potential for virus-laden particles to remain airborne for several hours and to penetrate deeply into the lungs. The implications of this research are not limited to hospital settings. Awareness of the aerosol transport of SARS-CoV-2 with fine PM may help to reduce transmission and support rationale for discouraging potential super-spreader events [28].

## Supplementary Information

**Additional file 1: Table S1.** Primers and probes used to target the nucleocapsid N gene^1^. **Table S2.** Patients in the emergency department (ED) and positive samples in the ED. **Figure S1. **Method for calculating copies/m^3^ from the qPCR copy number. A standard curve was used to estimate the number of copies per 2 µL of cDNA used in qPCR.

## Data Availability

The data that support the findings of this study are available from the corresponding author upon reasonable request with approval from the VA Boston Health Care System Research and Development Committee. Sequencing data that support the findings of this study have been deposited in GenBank with the accession number MW047086.

## Abbreviations

COVID-19: Coronavirus disease 2019
CW1: COVID-19 Ward 1
CW2: COVID-19 Ward 2
ED: Emergency department
GFF: Glass fiber filter
ICU: Intensive care unit
NCW: Non-COVID-19 ward
PM: Particulate matter
PPE: Personal protective equipment
PUF: Polyurethane foam
RT-qPCR: Reverse transcription quantitative polymerase chain reaction
SARS-CoV-2: Severe Acute Respiratory Syndrome Coronavirus 2
TSP: Total suspended particulate
