# Supplementary material for: Characterization of hospital airborne SARS-CoV-2
Source: Respir Res. 2021 Feb 26;22:73. doi: 10.1186/s12931-021-01637-8 (PMC7909372; doi:10.1186/s12931-021-01637-8)
Supplement: Supplementary file 1 — Additional file 1: Table S1. Primers and probes used to target the nucleocapsid N gene1. Table S2. Patients in the emergency department (ED) and positive samples in the ED. Figure S1. Method for calculating copies/m3 from the qPCR copy number. A standard curve was used to estimate the number of copies per 2 µL of cDNA used in qPCR. [file 12931_2021_1637_MOESM1_ESM.docx]

Supplementary Information

**Characterization of Hospital Airborne SARS-CoV-2**

Rebecca A. Stern^a^, Petros Koutrakis^b^, Marco A. G. Martins^b^, Bernardo Lemos^c^, Scot E. Dowd^d^, Elsie M. Sunderland^a,b^, Eric Garshick^e,f,g,*^

^a^Harvard John A. Paulson School of Engineering and Applied Science, Harvard University, Cambridge, MA, USA

^b^Department of Environmental Health, Harvard T.H. Chan School of Public Heath, Boston, MA, United States

^c^Department of Environmental Health & Molecular and Integrative Physiological Sciences Program, Harvard T.H. Chan School of Public Health, Boston, MA

^d^Molecular Research LP (MR DNA), Shallowater, TX, USA

^e^Pulmonary, Allergy, Sleep, and Critical Care Medicine Section, VA Boston Healthcare System, Boston, MA, USA

^f^Channing Division of Network Medicine, Brigham and Women’s Hospital, Boston, MA, USA

^g^Harvard Medical School, Boston, MA, USA

*Eric.Garshick@va.gov

**Table S1.** Primers and probes used to target the nucleocapsid N gene^1^

| **Name** | **Description** | **Oligonucleotide Sequence (5’>3’)** |
| --- | --- | --- |
| 2019-nCoV_N1-F | 2019-nCoV_N1 Forward Primer | GAC CCC AAA ATC AGC GAA AT |
| 2019-nCoV_N1-R | 2019-nCoV_N1 Reverse Primer | TCT GGT TAC TGC CAG TTG AAT CTG |
| 2019-nCoV_N1-P | 2019-nCoV_N1 Probe | FAM-ACC CCG CAT TAC GTT TGG TGG ACC-BHQ1 |
| 2019-nCoV_N1-P | 2019-nCoV_N1 Probe | FAM-ACC CCG CAT /ZEN/ TAC GTT TGG TGG ACC-3IABkFQ |
| 2019-nCoV_N2-F | 2019-nCoV_N2 Forward Primer | TTA CAA ACA TTG GCC GCA AA |
| 2019-nCoV_N2-R | 2019-nCoV_N2 Reverse Primer | GCG CGA CAT TCC GAA GAA |
| 2019-nCoV_N2-P | 2019-nCoV_N2 Probe | FAM-ACA ATT TGC CCC CAG CGC TTC AG-BHQ1 |
| 2019-nCoV_N2-P | 2019-nCoV_N2 Probe | FAM-ACA ATT TGC /ZEN/ CCC CAG CGC TTC AG-3IABkF |

**Table S2.** Patients in the emergency department (ED) and positive samples in the ED

| **Date** | **Total Respiratory Patients Evaluated in ED** | **Total ED Patients** | **Number of Positive Samples in ED** |
| --- | --- | --- | --- |
| April 29-May 1, 2020 | 12 | 55 | 0 |
| May 5-May 7, 2020 | 12 | 52 | 1 |
| May 11-May 13, 2020 | 22 | 63 | 0 |
| May 13-May 15, 2020 | 15 | 50 | 1 |
| May 18-May 20, 2020 | 20 | 64 | 0 |
| May 20-May 22, 2020 | 12 | 49 | 0 |

**Sampling location details**

Samplers were located: (1) in a corridor seven feet from the door to a negative pressure COVID-19 medical ward, (COVID-19 Ward 1, CW1); (2) in a 15-by-8 foot personal protective equipment (PPE) donning room located outside the entrance to a second negative pressure COVID-19 medical ward, directly in front of a station where persons put on PPE, including gowns, N95 masks, face shields, and shoe and head coverings (COVID-19 Ward 2, CW2); (3) in a corridor five feet from the entrance to the medical intensive care unit (requiring passage through a PPE donning room under negative pressure), (Intensive Care Unit, ICU); (4) a staff work station (an open area) of the emergency department eight feet from the entrance to two negative pressure rooms used for suspect COVID-19 patients (Emergency Department, ED), and; (5) at the staff work station of a ward that did not care for known COVID-19 patients (Non-COVID-19 Ward, NCW). This nurse’s station was in an open area with the nearest potential COVID-19 patients (up to 6 suspected patients) down a corridor with entry doors 25 feet away. Midway through the study period (on May 12-18, 2020), CW1 was closed and cleaned, then opened as a non-COVID-19 ward. This new ward included a negative pressure unit that cared for suspected COVID-19 patients in a smaller wing distant from the entrance.

**Sample collection details**

The cascade impactors were cleaned with 70% alcohol and assembled and disassembled aseptically inside a biosafety level 2 (BSL2) laminar flow hood. Subsequently, the cascades were attached to the top of the pump box (see Figure 2 in main paper). The sampling assemblies were sealed in plastic bags and transported aseptically to the hospital for sample collection. After sample collection, assemblies were sealed in plastic bags, and shortly thereafter were transported to and placed inside the BSL2 hood. Then, the cascade and pump box assemblies were removed from the plastic bags, cascades were detached from the box, and all box surfaces were sanitized with 70% ethanol. All personnel involved in sample collection, instrument setup and calibration, and sample processing wore personal protective equipment.

**Shotgun Sequencing**

Methods: One sample (VA-027-GFF) was selected at random out of the samples that amplified in PCR for shotgun sequencing to further assess the presence of SARS-CoV-2 RNA. The sample chosen was collected in the Emergency Department in the smallest particle size fraction on May 20-22, 2020. RNA quantity and quality were determined using a NanoDrop2000 (Thermo Scientific). 5 ul of RNA sample was first used for cDNA synthesis using QIAseq SARS-CoV-2 Primer Panel kit (Qiagen) followed by PCR using 2 pools of SARS-CoV-2 Primers. The PCR reaction was carried out with an initial holding stage of 98˚C for 2 minutes and 35 cycles of 98˚C for 20 seconds, followed by 65˚C for 5 minutes for annealing and extension. The PCR products obtained from 2 pools were pooled and purified using 1X PCR Clean DX beads (Aline Biosciences). The concentration of purified amplicon was evaluated using the Qubit® dsDNA HS Assay Kit (Life Technologies). The library was prepared using QIAseq FX DNA Library kits (Qiagen) following the manufacturer's user guide. 10 ng of purified amplicon was used to prepare the library. The sample underwent the fragmentation, end repair and A-addition followed by adapter ligation with unique indices. Library was amplified using adapter specific primers by following the manufacturer's user guide. Following the library preparation, the final concentration of the library was measured using the Qubit® dsDNA HS Assay Kit (Life Technologies), and the average library size was determined using the Agilent 2100 Bioanalyzer (Agilent Technologies). The library diluted (to 0.6nM) and sequenced paired end for 500 cycles using the NovaSeq system (Illumina).

Results: The sequenced SARS-CoV-2 RNA in sample VA-027-GFF was submitted to GenBank^2^ with accession number MW047086. Analysis via NextStrain^3^ produced multiple (conflicting) clade assignments, which suggests at least two distinct strains of SARS-CoV-2 in the sample. Sample VA-027-GFF previously had evidence of PCR amplification with a cycle threshold of 41.76, but it was above the 40.85 cutoff for positive samples that was determined based on the standard curve and a threshold of one copy of the virus per one microliter. Detection of SARS-CoV-2 in a sample that was near the PCR cycle threshold cutoff suggests that the detection of SARS-CoV-2 could have been underestimated in other samples.

**
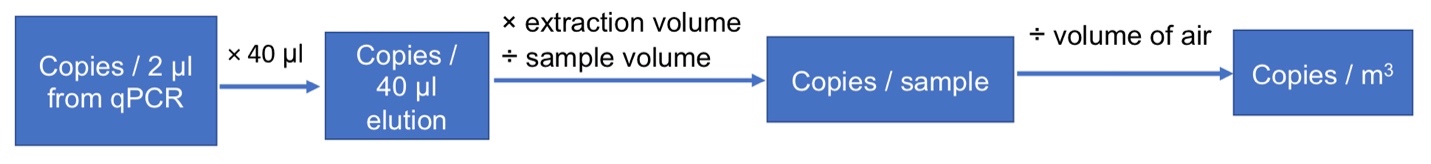
**

**Figure S1.** Method for calculating copies/m^3^ from the qPCR copy number. A standard curve was used to estimate the number of copies per 2 µL of cDNA used in qPCR.

**References**

1. US Centers for Disease Control and Prevention. 2019-Novel Coronavirus (2019-nCoV) Real-time rRT-PCR Panel Primers and Probes. https://www.cdc.gov/coronavirus/2019-ncov/downloads/rt-pcr-panel-primer-probes.pdf (2020).

2. Clark, K., Karsch-Mizrachi, I., Lipman, D. J., Ostell, J. & Sayers, E. W. GenBank. *Nucleic Acids Res.* **44**, D67–D72 (2016).

3. Hadfield, J. *et al.* Nextstrain: real-time tracking of pathogen evolution. *Bioinformatics* **34**, 4121–4123 (2018).
